# In broodmares undergoing artificial insemination is intrauterine fluid development more likely with frozen semen or chilled semen?

**DOI:** 10.18849/ve.v10i4.728

**Published:** 2025-12-04

**Authors:** Rumaysa Bint Saifullah+, Mary-Kate Burke+

**Keywords:** ARTIFICIAL INSEMINATION, ENDOMETRITIS, EQUINE, INTRAUTERINE FLUID, POSTBREEDING

## Abstract

**PICO question:**

In broodmares undergoing artificial insemination, is intrauterine fluid development more likely postinsemination with frozen semen compared to insemination with chilled semen?

**Clinical bottom line:**

**Category of research:**

Incidence.

**Number and type of study designs reviewed:**

Three retrospective cohort studies were critically appraised.

**Strength of evidence:**

Weak.

**Outcomes reported:**

A lower rate of postbreeding intrauterine fluid occurred in mares inseminated with frozen semen compared to chilled semen in two studies, and a higher rate in one study.

**Conclusion:**

The evidence available is weak and warrants further research into postbreeding intrauterine fluid rates in mares inseminated with frozen and chilled semen.

## How to apply this evidence in practice

The application of evidence into practice should take into account multiple factors, not limited to: individual clinical expertise, patient’s circumstances and owners’ values, country, location or clinic where you work, the individual case in front of you, the availability of therapies and resources.

Knowledge Summaries are a resource to help reinforce or inform decision-making. They do not override the responsibility or judgement of the practitioner to do what is best for the animal in their care.

## Clinical scenario

You are an equine veterinarian involved in assisted reproductive work during the breeding season. In order to improve pregnancy outcomes within your clinic, you seek to optimise postbreeding clinical management protocols for mares inseminated with chilled and frozen semen. To inform protocol development, you investigate the rate of postbreeding intrauterine fluid development in mares inseminated with frozen semen compared with those inseminated with chilled semen.

## The evidence

Three publications were found during the literature search that directly discussed the rates of postbreeding intrauterine fluid accumulation in mares inseminated with frozen and chilled semen, albeit not as the main clinical focus of the papers (Lewis et al., 2015; Crowe et al., 2008; Squires et al., 2006). Of these publications, all three were retrospective cohort studies of Level 4 status (Howick et al., 2011), or low evidence according to VetGRADE (Bowen et al., 2019).

Mares involved in the studies were of various ages, breeds, and reproductive statuses, although Lewis et al. (2015) used data predominantly from sporthorse mares. Multiple stallions contributed semen in all studies. Selection of either frozen or chilled semen was up to owner preference, resulting in sometimes vast differences in the number of mares inseminated with either type of semen.

Pre-insemination management was not standardised, with variations in insemination doses, number of doses, and timings of insemination. In addition, the timing of diagnosis of intrauterine fluid after insemination varied, and in Crowe et al. (2008) the timing remained unspecified. The parameters used to define postbreeding intrauterine fluid accumulation differed between the studies, varying from more than 1 cm of fluid (Crowe et al., 2008), more than 2 cm of fluid (Lewis et al., 2015), and any amount of fluid (Squires et al., 2006).

Statistical analysis of outcomes involving intrauterine fluid accumulation was performed in only one study (Lewis et al., 2015). Overall, two studies (Squires et al., 2006; Lewis et al., 2015) reported lower incidence proportions per cycle of postbreeding intrauterine fluid in mares inseminated with frozen as compared to chilled semen, with the final study (Crowe et al., 2008) reporting lower incidence proportions per cycle of postbreeding intrauterine fluid in mares inseminated with chilled compared to frozen semen. As such, the overall evidence indicates a lower incidence proportions per cycle of intrauterine fluid accumulation in mares inseminated with frozen semen. However, this evidence remains weak and calls for future research and investigation.

## Summary of the evidence

**Crowe et al. (2008):** A retrospective study of artificial insemination of 251 mares using chilled and fixed time frozen-thawed semen **Aim: **To compare the conception rate achieved by chilled and frozen semen artificial insemination, using fixed time insemination protocols over 2 breeding seasons.

Population:	Mares of a variety of breeds, ages (range 3–24 years) and reproductive status (maiden, barren, mares with foal at foot) from 3 stud farms.
Sample size:	251 mares.
Intervention details:	Mares were inseminated with either chilled or frozen semen using fixed-time artificial insemination (AI). Chilled semen protocol: Mares (n = 112) were implanted with deslorelin and inseminated 26–30 hours later, then checked for ovulation and had an exam of the uterus the next day. Single dose of chilled semen was given preovulation to 112 mares All mares ovulated after deslorelin administration. Frozen semen protocol: 2 doses (pre- and postovulation) of frozen semen were administered to the mares in this group (n = 139). 7 mares did not ovulate within expected 48 hour window; 2 of these 7 then ovulated within 54 hours. Second deslorelin implant given to the remaining 5 mares who had not ovulated by 60 hours. Mares in both groups received the same post insemination management: All mares received oxytocin, uterine irrigation, sodium benzylpenicillin, and framycetin intrauterine after insemination. If > 1 cm intrauterine fluid was present 24 hours postinsemination, then uterine lavage with 1 L sterile saline was performed and the exam was repeated next day.
Study design:	Retrospective cohort study.
Outcome Studied:	Comparing conception rates between chilled and frozen semen.
Main Findings (relevant to PICO question):	Postinsemination fluid rate higher for frozen semen (60/139, 43.4%) than chilled semen (40/112, 35.7%). Pregnancy rate for mares with postinsemination fluid higher for frozen semen (114/139, 82%) than chilled semen (78/112, 69.6%).
Limitations:	Retrospective in nature so no control group or randomisation. Lack of statistical analysis in relation to postinsemination fluid. A variety of factors could have influenced the formation of postbreeding endometritis, e.g. age. The effect of these mare factors was not analysed. Non-standardised artificial insemination protocols used for both chilled and frozen semen, including site of insemination, dose volume and number of doses. Owner preference regarding choice of semen increases the external validity of the study while reducing the internal validity.

**Lewis et al. (2015):** Utilization of One-Dose Postovulation Breeding With Frozen-Thawed Semen at a Commercial Artificial Insemination Center: Pregnancy Rates and Postbreeding Intrauterine fluid Accumulation in Comparison to Insemination With Chilled or Fresh Semenintrauterine fluid **Aim: **To determine the pregnancy rates and incidence of postbreeding intrauterine fluid associated with insemination of mares with frozen semen.

Population:	Predominantly sport-horse mares of varied reproductive history aged 2–27 years from a single artificial insemination centre in the UK.
Sample size:	578 mares (1023 cycles).
Intervention details:	Chilled semen (n = 242 cycles) was inseminated into the uterine body 24 hours postadministration of ovulation induction agent (Human Chorionic Gonadotropin (hCG) or deslorelin), i.e. preovulation. Only cycles where ovulation occurred within 24 hours of insemination were included in the study. Frozen semen (n = 459 cycles) was inseminated postovulation. Postbreeding management was unique to each mare: Same-day lavage performed if history of susceptibility to intrauterine fluid accumulation or indications of potential susceptibility (e.g. higher age/parity). Year 1: All mares receiving frozen semen thought to be at greater risk of developing intrauterine fluid were lavaged postinsemination. Year 2: Criteria for lavage in frozen semen mares was more stringent. Year 3: Selection criteria for lavage of frozen semen mares was the same as for chilled or fresh semen. All mares examined 24 hours postinsemination. If > 2 cm intrauterine fluid was present lavage was repeated for 1–3 days with oxytocin +/- intrauterine antibiotics.
Study design:	Retrospective cohort study.
Outcome Studied:	Incidence proportions per cycle of postbreeding intrauterine fluid accumulation following the use of frozen semen as outlined above. Pregnancy rates for chilled semen and single-dose post ovulation insemination of frozen semen. Impact of prophylactic postinsemination lavage.
Main Findings (relevant to PICO question):	Overall incidence proportions per cycle of > 2 cm postbreeding fluid was 6.3% (29/459) for frozen and 17.8% (43/242) for chilled semen. Incidence proportions per cycle of > 2 cm postbreeding fluid was significantly lower with same-day lavage for frozen semen (5.6%) as compared to chilled semen (11%). Use of uterine lavage decreased over the 3 years but incidence proportions per cycle of > 2 cm intrauterine fluid remained the same over the 3 years. Odds of > 2 cm detectable postbreeding intrauterine fluid were significantly less when frozen semen was used compared with chilled semen, after adjusting for mare age, status, and management factors.
Limitations:	Retrospective in nature so no control group or randomisation. Non-standardised artificial insemination protocols used for both chilled and frozen semen. Owner preference regarding choice of semen increases the external validity of the study while reducing the internal validity. Varied reproductive histories reduce the internal validity of the study. Non-standardised management for postbreeding fluid throughout the years particularly the use of same-day lavage.

**Squires et al. (2006):** Retrospective study of factors affecting fertility of fresh, cooled and frozen semen **Aim: **To determine factors which affect the fertility of mares inseminated with fresh, chilled, and frozen semen and compare the pregnancy rates of these mares.

Population:	Mares from 6 different facilities in Maryland, Kentucky, Florida, Texas, Italy and Germany; no breed specified.
Sample size:	961 mare cycles examined over two breeding seasons: 2002 (407 cycles) and 2003 (554 cycles).
Intervention details:	Data was collected retrospectively from 6 different facilities in the USA and Europe and was sent to Colorado State Univeristy for Chi-squared data analysis Mares were inseminated with semen from a variety of different sources.
Study design:	Retrospective cohort study.
Outcome Studied:	Effects of mare age, reproductive status, sperm type, insemination frequency, and timing of insemination on pregnancy rates.
Main Findings (relevant to PICO question):	Incidence proportions per cycle of postbreeding fluid: cooled 32%, frozen 23% (full dataset unavailable). Multiple inseminations with frozen semen did not increase incidence proportions per cycle of intrauterine fluid or decrease fertility. The results of the fresh semen will not be discussed further as they were not relevant to the PICO.
Limitations:	Retrospective in nature so no control group or randomisation. Incidence proportions per cycle percentages reported of postbreeding fluid were only stated in the discussion and not formally presented or discussed in the results section. Hence the data associated with these percentages is not accessible. No statistical analysis of postbreeding fluid results. Potential inconsistencies with multiple operators measuring and treating intrauterine fluid. No defined criteria for intrauterine fluid accumulation.

## Appraisal, application and reflection

Postbreeding endometritis is the mare’s normal inflammatory reaction to semen. It occurs before the embryo reaches the uterus, which is usually about 5.5 days after fertilisation (LeBlanc & Causey, 2009). This results in accumulation of intrauterine fluid, which provides a poor environment for embryonic development. It is a recognised cause of reduced pregnancy rates in mares and is typically managed through a combination of ecbolic agents, uterine lavage, intrauterine mucolytics, systemic or intrauterine anti-inflammatories, and/or systemic or intrauterine antibiotics (LeBlanc & Causey, 2009).

The rates of development of intrauterine fluid using frozen as compared to chilled semen have not been widely documented in the literature. The popularity of frozen semen has generally been on the rise (Kowalczyk et al., 2019). Characterising differences in the incidence proportions per cycle of postbreeding endometritis using frozen and chilled semen may help us to improve the clinical management of mares after a service, with the aim to improve pregnancy rates.

Postbreeding intrauterine fluid in mares is diagnosed and classified with ultrasound, and can be difficult for the clinician to manage. Due to its associations with reduced pregnancy rates, it must be cleared effectively to create a uterine environment that is conducive to embryonic growth (Maischberger et al., 2008). It is currently hypothesised that seminal plasma plays an important role in suppressing and modulating the postbreeding inflammatory response (Troedsson et al., 2001; Maischberger et al., 2008), thus reducing the risk of intrauterine fluid buildup. In the process of cryopreservation, seminal plasma is removed, and cryopreservation agents are added (Bubenickova et al., 2020). As such, it is suggested that persistent postbreeding endometritis may occur more commonly in mares inseminated with frozen semen (Troedsson et al., 2001).

Only three studies (Crowe et al., 2008; Lewis et al., 2015; Squires et al., 2006) were identified that addressed the development of postbreeding intrauterine fluid following administration of frozen and chilled semen. There appears to be no standard explicit definition of intrauterine fluid accumulation across these papers: Crowe et al. (2008) defined it as > 1 cm fluid, Lewis et al. (2015) as > 2 cm fluid, and Squires et al. (2006) as any amount of fluid. As such, any conclusions drawn must take into account that some horses with postbreeding intrauterine fluid in Crowe et al. (2008) and Squires et al. (2006) may have been excluded from these studies, thus potentially influencing the outcomes of the studies. In addition, there is a risk of human error with regard to measurements made on ultrasound, and as such any borderline measurements (e.g. close to 1 cm or close to 2 cm) may have been excluded or included.

Two of the three studies concluded that postbreeding intrauterine fluid was lower when using frozen semen compared to chilled semen (Lewis et al., 2015; Squires et al., 2006); the third study concluded the postbreeding intrauterine fluid was lower when using chilled semen compared to frozen semen (Crowe et al., 2008). Of the three studies, only Lewis et al. (2015) performed statistical analysis of these results, determining that the difference was significant; it is difficult to make conclusions about the significance of the results from the other two papers. The postinsemination management protocol in Lewis et al. (2015) changed over three years of measurements, particularly with regards to postbreeding uterine lavage which was reduced over the years; this may have influenced the incidence proportions per cycle of mares with intrauterine fluid. Despite these changes, the incidence proportions per cycle of intrauterine fluid reportedly remained unchanged over the three years. The odds of detecting > 2 cm postbreeding intrauterine fluid were significantly lower with frozen semen as compared to chilled semen, and the overall incidence proportions per cycle also lower (6.3% (29/459) for frozen, 17.8% (43/242) for chilled). Interestingly, pregnancy rates were similar for frozen (48.6%) and chilled (43%) semen mares. The lower percentage of intrauterine fluid with frozen semen may have led to higher pregnancy rates than normal. A possible reason for the similar pregnancy rate in chilled semen may be a reflection of the higher pregnancy rates reported in the literature using chilled semen as compared to frozen semen (Troedsson et al., 2001; Kelly, 2022). However, it must be noted that comparison of pregnancy rates between the studies is difficult, as different measurement units were used by each study: Crowe et al. (2008) used mares as the unit, Squires et al. (2006) used cycles and Lewis et al., (2015) used both mares and cycles.

All three studies used a large population of mares of varying breeds, ages, and reproductive statuses, with an exception in Lewis et al. (2015), which used data from predominantly sport-horse mares. Although this makes data extrapolation to the general equine population somewhat more reliable, there is a failure to account for other factors which may affect fertility of the mare, such as age in Crowe et al. (2008) and Squires et al. (2006). Squires et al. (2006) recorded a lower incidence proportions per cycle of postbreeding intrauterine fluid in a group of mares inseminated with frozen semen (23%) as compared to chilled semen (32%). However, it was also noted that, for the chilled semen group, there was a significantly lower fertility associated with older mares (ages 10+ years) than with those younger than 10 years old. In contrast, there was no significant effect of age associated with fertility for mares inseminated with frozen semen. Lewis et al. (2015) also reported no significant effect of age associated with fertility for mares inseminated with frozen semen. This study used multivariable model which included mare age as a fixed effect and mare as a random effect, and adjusted for factors such as reproductive status and management. The study concluded that the odds of detecting > 2 cm intrauterine fluid remained significantly lower for frozen semen than chilled semen.

The insemination protocol was not standardised across the studies, and thus may have influenced the incidence proportions per cycle of postbreeding intrauterine fluid. Lewis et al. (2015) described one preovulatory insemination dose for chilled semen and one postovulatory dose for frozen semen. The dataset in Squires et al. (2006) was more variable, with mares inseminated either once or with a combination of pre- and/or postovulation inseminations. Finally, the protocol in Crowe et al. (2008) consisted of one preovulation dose for chilled semen and two doses (one preovulation and one postovulation) for frozen semen. Theoretically, multiple doses of semen may cause a more severe inflammatory response and thus a reduced likelihood of pregnancy. However, Crowe et al. (2008) reported the best pregnancy rates for the use of frozen semen. Squires et al. (2006) also reported no significant difference in pregnancy rates with frozen semen (despite the number of inseminations), in contrast to chilled semen. In addition, pregnancy rates in mares inseminated multiple times were higher for chilled semen mares, but not for frozen semen. The lack of standardisation of insemination protocols and interventions for postbreeding intrauterine fluid makes comparisons between the studies and, hence, the ability to draw conclusions more challenging.

The strength of the current evidence with regard to the PICO question is weak. Overall, the evidence for the rates of intrauterine fluid accumulation in mares inseminated with chilled compared to frozen semen is conflicting, with arguments available for higher rates using both types of semen, although the Lewis et al. (2015) would suggest lower rates for frozen semen. It could be argued that the most recent study (Lewis et al., 2015) is more representative, as it had the largest number of mare cycles studied. Statistical analysis was also performed in this study which increases the scientific rigour of the study and in turn the usefulness of the data to clinicians. However, there are limitations that need to be accounted for when interpreting the results. Future work should ideally include prospective, blinded studies with more rigorous inclusion criteria to allow for control over other factors influencing fertility and the development of postbreeding intrauterine fluid. This may be difficult to achieve due to the emotional and financial investments in the equine breeding industry.

## Methodology

**Search Strategy:** 

Databases searched and dates covered:	CAB Abstracts on OVID (1970–2025) PubMed on OVID (1970–2025) BEVA on Wiley Online Library (1970–2025)
Search strategy:	CAB Abstracts on OVID: (equine OR horse OR mare OR broodmare) AND (artificial insemination OR AI) AND ((frozen OR thawed) AND (chilled OR cooled)) AND (endometritis OR post breeding endometritis OR intrauterine fluid OR post breeding fluid OR uterine fluid) PubMed on National Library of Medicine: (equine OR horse OR mare OR broodmare) AND (artificial insemination OR AI) AND ((frozen OR thawed) AND (chilled OR cooled)) AND (endometritis OR post breeding endometritis OR intrauterine fluid OR post breeding fluid OR uterine fluid) BEVA Wiley Online Library: (equine OR horse OR mare OR broodmare) AND (artificial insemination OR AI) AND (endometritis OR post breeding endometritis OR intrauterine fluid OR post breeding fluid OR uterine fluid) AND ((frozen OR thawed) AND (chilled OR cooled))
Dates searches performed:	24 Apr 2025

**Exclusion / Inclusion Criteria:** 

Exclusion:	Non-English language, review articles, case reports, conference proceedings/abstracts.
Inclusion:	Retrospective studies, multi-centre studies, single-centre studies, experimental studies.

**Search Outcome:** 

Database	Number of results	Excluded – did not answer the PICO question	Excluded – review articles/book chapters/editorials, etc.	Excluded – non-English language	Total relevant papers
CAB Abstracts	58	49	7	0	2
PubMed	7	7	0	0	0
BEVA	20	19	0	0	1
Total relevant papers when duplicates removed					3

## ORCiD

Rumaysa Bint Saifullah: https://orcid.org/0009-0001-9234-7233

Mary-Kate Burke: https://orcid.org/0009-0003-5322-3814

## Conflict of Interest

The author declares no conflicts of interest.
